# INHBB is a novel prognostic biomarker and correlated with immune infiltrates in gastric cancer

**DOI:** 10.3389/fgene.2022.933862

**Published:** 2022-09-02

**Authors:** Weifeng Yu, Guihua He, Wang Zhang, Zhenhao Ye, Zishao Zhong, Suiping Huang

**Affiliations:** ^1^ Gastroenterology Department, The Second Affiliated Hospital of Guangzhou University of Chinese Medicine, Guangzhou, China; ^2^ Gastroenterology Department, Guangdong Provincial Hospital of Chinese Medicine, Guangzhou, China; ^3^ State Key Laboratory of Dampness Syndrome of Chinese Medicine, The Second Affiliated Hospital of Guangzhou University of Chinese Medicine, Guangzhou, China

**Keywords:** gastric cancer, prognostic marker, INHBB, immune cell infiltration, the cancer genome atlas, genotype-tissue expression

## Abstract

Inhibin subunit beta B (INHBB) is a potential prognostic biomarker for a variety of cancers. However, its role in gastric cancer (GC) remains elusive. The differential expression data of *INHBB* in tumor and normal tissues were extracted from several databases and genetic alterations of *INHBB* were assessed by cBioPortal. Kaplan-Meier analysis was used to evaluate the survival rate of patients with GC with *INHBB* and association with clinical features in GC. Cox regression analysis was used to explore the prognostic value of clinical indicators and *INHBB* in GC, and a nomogram prognostic model was established. In addition, the predictive validity of the nomogram model was assessed by time-depended receiver operating characteristic (ROC) and calibration curves. Functional enrichment analyses were conducted to functionally annotate *INHBB*. Notably, we found that the quantitative assessment of immune cell subpopulation infiltration correlated with *INHBB* expression. *INHBB* expression is upregulated in GC and is correlated with several clinical features including prognostic indicators and a histological type. Genetic alterations were observed in *INHBB*, its DNA methylation level was negatively correlated with *INHBB* expression. High *INHBB* expression is associated with a poor prognosis and is an independent risk factor for prognosis in GC, along with age and residual tumor. The nomogram model showed a good prediction ability and was validated by time-depended ROC and calibration curves. Functional enrichment analysis indicated that *INHBB*-associated genes were enriched in tumor microenvironment Gene Ontology (GO) terms and were correlated with tumor-associated pathways. INHBB has a regulatory function in immune cell infiltration, especially macrophage infiltration in GC. Specifically, patients with GC with high *INHBB* expression and high macrophage infiltration have a worse prognosis. *INHBB* expression was negatively correlated with the expression of chemokines/chemokine receptors and plays a regulatory role in immunoinhibitor/immunostimulator-involved pathways. INHBB is a potential prognostic biomarker for GC and may drive the abnormal activity of critical cancer-associated pathways, potentially contributing to immune cell infiltration to promote GC development.

## Introduction

Gastric cancer (GC) is a malignant tumor originating from the epithelium of the gastric mucosa. It ranks fifth in incidence and third in mortality among all tumors, with nearly 800,000 patients dying of GC worldwide in 2018 (Bray et al., 2018). The primary treatment in GC involves surgical resection of target lesions, followed by adjuvant chemotherapy (Sohn et al., 2017). Recently, advances in treatment such as inhibition of immune checkpoint or cancer stemness have changed the prognosis of patients with early GC. However, the overall survival rate is still unsatisfactory (Smyth et al., 2020), this may be attributed to occult development and non-specific clinical symptoms, and there is a lack of reliable specific markers. Traditional prognostic markers lack sensitivity because of which the patients with GC lose the early-stage treatment opportunities to treat malignant invasions, which may later develop into metastasis leading to a poor prognosis (Dicken et al., 2005). Therefore, it is pivotal to find reliable prognosis indicators to promote GC patient survival.

Activins, members of the transforming growth factor TGF-β superfamily of proteins, are synthesized as homo- or hetero-dimers of two highly related disulfide-linked inhibin beta subunits, inhibin subunit beta A (encoded by INHBA) and inhibin subunit beta B (encoded by INHBB). Thus, INHBA and INHBB can form three molecular species activin A, activin B, and activin AB (Chang et al., 2002; Rodgarkia-Dara et al., 2006). Activins are widely expressed and are functionally diverse; for example, they are involved in inflammation, cellular proliferation, and embryogenesis (Ethier and Findlay, 2001; Werner and Alzheimer, 2006; Xia and Schneyer, 2009). While the beta subunits have a similar sequence identity, the potency of each subunit in regulating biological processes shows a considerable variation (Muttukrishna et al., 1994). In the past, most research on INHBB focused on the reproductive system (Tong et al., 2003; Yao et al., 2006). Recently, INHBB was regarded as a valuable biomarker in various cancer types. Gutierrez et al. demonstrated that INHBB is regulated by methylation and closely associated with metastasis in colorectal cancer (Gutierrez et al., 2021). Xu et al. (2020) indicated that INHBB expression is upregulated in rectal cancer tissues and portends a poor prognosis. Moreover, Kita, Akihiro et al. suggested that high INHBB expression promotes cell migration and proliferation in oral squamous cell carcinoma and is associated closely with the tumor microenvironment (Kita et al., 2017). All these data suggested that INHBB may be a novel oncogene and associated with tumor progression, but the role of INHBB in GC remains unknown.

In this pilot study, we aimed to investigate the expression of INHBB in The Cancer Genome Atlas (TCGA) and Gene Expression Omnibus (GEO) databases and explore the prognostic value of INHBB in GC. Further, we verified the results using a GC clinical tissue microarray. The association between INHBB and immune infiltrates was also explored through bioinformatic analyses. In addition, correlation analyses between INHBB expression and immunomodulators as well as chemokines were performed to identify the potential immunoregulation of INHBB in GC.

## Materials and methods

### Data collection and analysis

INHBB expression data of the Genotype-Tissue Expression (GTEx) and TCGA were obtained from the UCSC Xena database (https://xenabrowser.net/datapages/) and have been uniformly processed using the Toil process (Vivian et al., 2017). Meanwhile, the GC data in TCGA and the corresponding healthy stomach tissue data in GTEx were extracted for further analyses. A scatter plot was used to show the difference in expression of INHBB in tumor and healthy tissues. The GSE26899 and GSE29272 datasets contained 108 and 268 tissue expression profiling samples, respectively, with a total of 146 normal samples and 230 gastric cancer samples. It was obtained from the GEO database (https://www.ncbi.nlm.nih.gov/geo/) to validate the expression of INHBB in GC.

### Genetic alterations of inhibin subunit beta B

The cBioPortal for Cancer Genomics (http://cbioportal.org) (Gao et al., 2013) is a comprehensive genomics database which is widely used in the cancer field. Using cBioPortal, we visualized the genetic variation of INHBB in GC. Illumina HumanMethylation 450 BeadChip is a new platform with 450,000 DNA methylation detection sites; methylation profiles were downloaded from Illumina human methylation 450 in TCGA and used for correlation analysis with INHBB expression data.

### Survival analysis

R package survival was used to analyze the overall survival of patients with high or low INHBB expression in GC using pan-cancer level data from TCGA which was visualized using the survminer package (v.0.4.9). Kaplan-Meier plotter (www.kmplot.com) (Gyorffy, 2021) was used to assess the prognostic value of INHBB in GC data from the GEO database (GSE15459, GSE29272, GSE62254). We also explored the role of macrophage infiltration levels in the prognosis of patients with GC in the pan-cancer module.

### Functional enrichment analysis

Gene Ontology (GO) and Kyoto Encyclopedia of Genes and Genomes (KEGG) analyses were performed by R package org. Hs.eg.db (Bioconductor 3.2) and clusterProfiler (v.3.6.3) (Yu et al., 2012), and the result was visualized using ggplot2 (v.3.3.3). Gene set enrichment analysis (GSEA) was analyzed based on Hallmark gene set terms, the false discovery rate (FDR) q-value, normalized enrichment score (NES), and nominal *p*-value suggested the importance of the correlation between gene sets and pathways. Gene sets with *p* value <0.05 and FDR <0.25 were considered as significantly enriched.

### Immune cell infiltration analysis

Gene set variation analysis (GSVA, v.1.34.0) was used to demonstrate the correlation of infiltration of 24 types of immune cells with INHBB expression in GC. Tumor Immune Estimation Resource (TIMER, https://cistrome.shinyapps.io/timer/), includes the data of 10,897 tumors from 32 cancer types and is a comprehensive analytical web tool to explore the molecular interactions of tumor immune infiltration and gene expression data (Li et al., 2017). Gene Expression Profiling Interactive Analysis (GEPIA, http://gepia.cancer-pku.cn/index.html) is an online platform application for gene expression analysis based on the data from the TCGA and the GTEx databases (Tang et al., 2017). We thus explored the association between INHBB and tumor-infiltrating immune cells. Meanwhile, we visualized six types of immune cell infiltration via TIMER and uncovered the prognostic impact of immune infiltrates in GC. Additionally, we visualized six types of immune cells involved in infiltration *via* TIMER and uncovered the prognostic impact of immune infiltrates in GC. We further explored the correlation between INHBB expression and immune cell markers using TIMER and GEPIA.

### Immunomodulator analysis

TISIDB (http://cis.hku.hk/TISIDB/index.php) is a publicly open website for assessing tumor and immune system interaction (Ru et al., 2019). In the immunomodulator and chemokine modules, we explored the association between INHBB expression and immunoinhibitors/immunostimulators as well as chemokines/chemokine receptors at the GC and pan-cancer levels.

### Clinical materials

GC tissue microarray samples from 97 patients with GC and paracarcinoma tissues from 83 patients were obtained from Shanghai Outdo Biotech (HStmA180Su08). According to the seventh edition of the UICC/AJCC TNM staging system, the patients were divided into different tumor clinical stages. All these patients were diagnosed with primary GC and they underwent surgery from December 2013 to September 2015, with a 5-year follow-up.

### Immunohistochemistry

Microarray analysis of GC tissues and matched paracarcinoma tissues was performed. For Immunohistochemistry (IHC), the microarray samples were stained with an INHBB antibody (Invitrogen, PA5-119792). The steps are briefly described as follows. After paraffin-embedded sections of GC tissue specimens were dewaxed and hydrated with xylene as well as ethanol at different gradient concentrations, microwave antigen repair was performed, followed by incubation with hydrogen peroxide solution at room temperature for 10 min to block endogenous peroxidase activity. The primary antibody was added and incubated overnight at 4°C, washed three times with phosphate-buffered saline, and incubated with a secondary antibody at room temperature for 30 min. The sections were stained with 3,3′-diaminobenzidine (DAB) followed by hematoxylin re-staining, and finally, the slices were sealed with neutral gum.

The staining intensity and positive staining rate of cytoplasmic staining of INHBB were calculated independently for cancer and paracarcinoma tissues. The immunostaining index was based on the proportion of positively stained tumor cells and staining intensity. The proportion of positive staining rate was set from 0 to 100%, and the staining intensity score was divided into four categories: 0 (no immunostaining), 1 (weak), 2 (moderate), and 3 (strong). The staining intensity score multiplied by the staining positive score was finally calculated as the immunostaining index; tumors with indexes less than or equal to 140% were considered as immunostaining-low expression and those more than 140% were scored as immunostaining-high expression.

### Statistical analysis

All data analyses were performed using R (v.3.6.3). A median threshold was employed to distinguish between high and low expression of INHBB. The association between INHBB expression and clinical pathologic variables was analyzed using Wilcoxon rank-sum, Chi-square, and Fisher’s exact tests. The Kaplan–Meier curve was used to estimate survival rates between the high and low INHBB-expressing groups. Univariate and multivariate Cox regression analyses were used to identify the importance of prognostic factors. *p*-values less than 0.05 were considered statistically significant.

## Results

### Inhibin subunit beta B is highly expressed in gastric cancer

**FIGURE 1:**
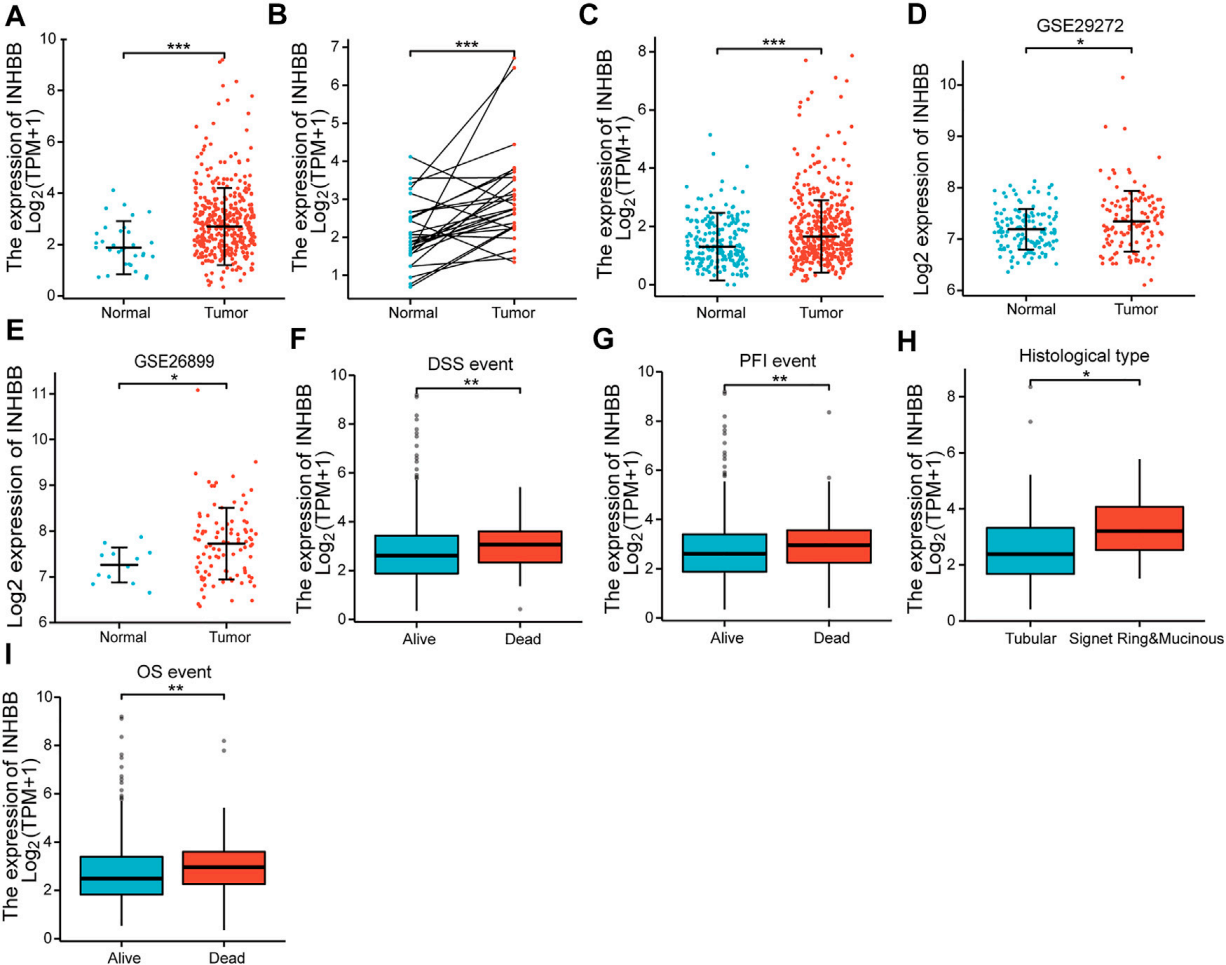
The expression of INHBB in **(A)** GC tissues and normal tissues data from TCGA, **(B)** GC tissues and paired paracarcinoma tissues data from TCGA, **(C)** GC tissues and normal tissues data from TCGA and GTEx, **(D,E)** GC tissues and normal tissues data from GSE29272 and GSE26899, and the association between INHBB expression and **(F)** DSS event, **(G)** PFI event, **(H)** histological type, **(I)** OS event.

First, we evaluated the mRNA expression levels of INHBB in patients with GC. The results suggested that INHBB expression in GC tissues was significantly higher than that in normal tissues (*p* < 0.001) (Figure 1A). The results were verified in GC and paired adjacent normal tissues (*p* < 0.001) (Figure 1B). The same results were obtained during the combined analysis of normal tissues in TCGA and GTEx databases (Figure 1C). In addition, we downland two GEO datasets (GSE26899, and GSE29272) to validate the transcription expression level of INHBB in cancer tissues and adjacent tissues. The results indicated that the expression of INHBB in GC lesions was significantly higher than that in adjacent noncancerous tissues from GEE26899 and GSE29272 datasets (*p* < 0.05) (Figures 1D, E).

Next, we analyzed the association between clinical characteristics and INHBB expression in patients with GC in the TCGA database. The clinical categories of patients with GC are summarized in Table 1, for example, it is clearly shown that the frequencies of T4 stage GC patients are 13.1% and 14.2% in low and high expression groups, respectively. The results indicated that high INHBB expression was significantly correlated with the histological type (*p* < 0.05), OS event (*p* < 0.01), DSS event (*p* < 0.01), and PFI event (*p* < 0.01) (Figures 1F–I). These results show that INHBB is significantly upregulated in GC and correlated with patient clinical characteristics.

**TABLE 1:** Clinical characteristics of patients with GC based on TCGA.

Characteristic		Low expression of INHBB		High expression of INHBB		p
		N	%	N	%	
T stage	T1	15	4.1%	4	1.1%	0.060
	T2	41	11.2%	39	10.6%	
	T3	78	21.3%	90	24.5%	
	T4	48	13.1%	52	14.2%	
N stage	N0	56	15.7%	55	15.4%	0.841
	N1	48	13.4%	49	13.7%	
	N2	40	11.2%	35	9.8%	
	N3	34	9.5%	40	11.2%	
M stage	M0	165	46.5%	165	46.5%	1.000
	M1	13	3.7%	12	3.4%	
Pathologic stage	Stage I	32	9.1%	21	6%	0.228
	Stage II	48	13.6%	63	17.9%	
	Stage III	76	21.6%	74	21%	
	Stage IV	19	5.4%	19	5.4%	
Primary therapy outcome	PD	32	10.1%	33	10.4%	0.946
	SD	7	2.2%	10	3.2%	
	PR	2	0.6%	2	0.6%	
	CR	112	35.3%	119	37.5%	
Gender	Female	70	18.7%	64	17.1%	0.564
	Male	117	31.2%	124	33.1%	
Age	≤65	83	22.4%	81	21.8%	0.880
	>65	102	27.5%	105	28.3%	
Histological type	Diffuse type	32	8.6%	31	8.3%	**0.045**
	Mucinous type	6	1.6%	13	3.5%	
	Not otherwise specified	105	28.1%	102	27.3%	
	Papillary type	1	0.3%	4	1.1%	
	Signet ring type	2	0.5%	9	2.4%	
	Tubular type	41	11%	28	7.5%	
Residual tumor	R0	147	44.7%	151	45.9%	0.443
	R1	6	1.8%	9	2.7%	
	R2	10	3%	6	1.8%	
Histologic grade	G1	3	0.8%	7	1.9%	0.393
	G2	72	19.7%	65	17.8%	
	G3	110	30.1%	109	29.8%	
Anatomic neoplasm subdivision	Antrum/Distal	76	21.1%	62	17.2%	0.098
	Cardia/Proximal	21	5.8%	27	7.5%	
	Fundus/Body	59	16.3%	71	19.7%	
	Gastroesophageal junction	18	5%	23	6.4%	
	Other	4	1.1%	0	0%	
Reflux history	No	100	46.7%	75	35%	0.844
	Yes	21	9.8%	18	8.4%	
Antireflux treatment	No	81	45.3%	61	34.1%	0.888
	Yes	20	11.2%	17	9.5%	
Barretts esophagus	No	114	54.8%	79	38%	0.871
	Yes	8	3.8%	7	3.4%	
*H pylori* infection	No	82	50.3%	63	38.7%	0.782
	Yes	9	5.5%	9	5.5%	
OS event	Alive	130	34.7%	98	26.1%	**< 0.001**
	Dead	57	15.2%	90	24%	
DSS event	Alive	141	39.8%	122	34.5%	**0.004**
	Dead	32	9%	59	16.7%	
PFI event	Alive	138	36.8%	113	30.1%	**0.007**
	Dead	49	13.1%	75	20%	

Bold: N, number; %, percentage; p, *p*-value

**FIGURE 2:**
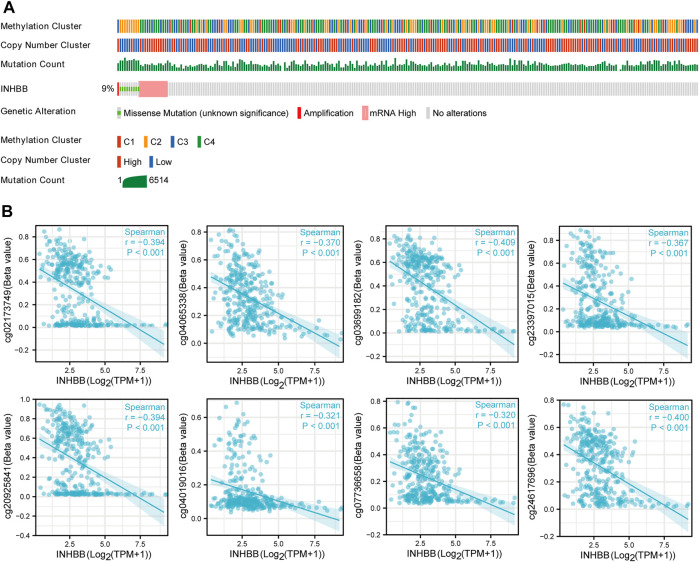
The epigenetic changes of INHBB in GC. **(A)** Genetic alteration of INHBB in GC, **(B)** Correlation of methylation and INHBB expression in GC.

To further clarify the potential mechanisms of abnormal INHBB expression, we explored the mutation alteration of INHBB in GC by using cBioPortal databases, as the result showed that INHBB had less than 9% missense mutations and gene amplifications in GC (Figure 2A). Furthermore, methylation data in TCGA databases were analyzed to evaluate the pre-transcriptional modification status of INHBB in GC. The results showed that the methylation levels were negatively correlated with INHBB expression in GC (Figure 2B).

### High inhibin subunit beta B expression correlated to poor prognosis in gastric cancer

In order to identify the effect of INHBB expression on patient survival, we divided patients with GC into two groups of high and low expression by the mean expression value of INHBB. The results of Kaplan-Meier analysis indicated that high INHBB expression was related to a poor prognosis of overall survival (OS) in seven cancer types, which is contrary to that in THCA, including BLCA, COAD, GBMLGG, HNSC, COADREAD MESO and UVM (Supplementary Figure S1). Next, we explored the relationship between high INHBB expression and disease-specific survival (DSS) as well as progression-free survival (PFI). The data showed that upregulated expression of INHBB has a worse DSS in BLCA, COAD, GBMLGG, COADREAD, KIRP, LIHC, UVM (Supplementary Figure S2) and a worse PFI in COAD, COADREAD, LUSC, UVM (Supplementary Figure S3).

**FIGURE 3:**
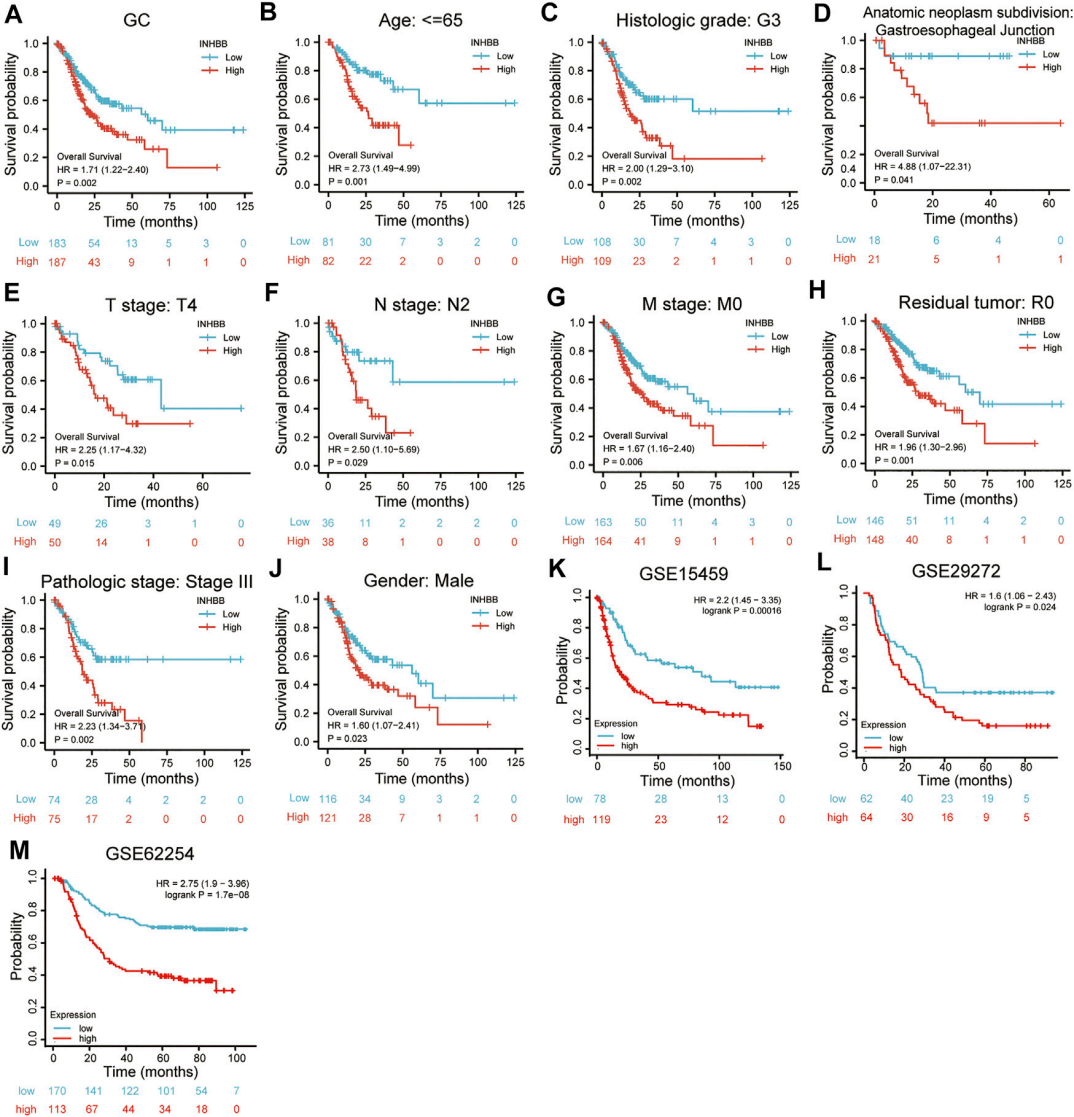
Kaplan-Meier survival analysis of INHBB expression and **(A)** prognosis in GC patient data from TCGA, **(B)** age, **(C)**histological grade, **(D)** anatomic neoplasm subdivision, **(E)** T stage, **(F)** N stage, **(G)** M stage, **(H)** residual tumor, **(I)** pathologic stage, **(J)** gender, prognosis in GC data from **(K)** GSE15459, **(L)** GSE29272, **(M)** GSE62254.

In patients with GC, high INHBB expression correlated with poor OS (HR = 1.71, *p* = 0.002) (Figure 3A). Subgroup analysis indicated that high expression of INHBB was significantly correlated with poor OS in GC in the following categories: patients less than 65 years old (HR = 2.73, *p* = 0.001), male patients (HR = 1.60, *p* = 0.023), T4 (HR = 2.25, *p* = 0.015), N2 (HR = 2.5, *p* = 0.029), M0 (HR = 1.67, *p* = 0.006), G3 (HR = 2.00, *p* = 0.002), pathological stage Ⅲ (HR = 2.23, *p* = 0.002), residual tumor R0 (HR = 1.96, *p* = 0.001), and those with gastroesophageal junction (HR = 4.88, *p* = 0.041). These data are shown in Figures 3B–J. Simultaneously, we conducted a Kaplan–Meier survival analysis of DSS and PFI to identify the prognostic role of INHBB in GC; the results showed that INHBB expression correlated with adverse prognosis of PFI (HR = 1.66, *p* = 0.006) and DSS (HR = 2.08, *p* = 0.001), subgroup analysis of DSS and PFI showed that T3/T4, N2, M0, R0, G3, age less than 65 years old, no barretts esophagus, no reflux history, and anatomic neoplasm subdivision cardia/proximal are associated poor DSS and PFI in GC (Supplementary Figures S4, S5).

Furthermore, we used three GEO datasets (GSE15459, GSE29272, and GSE62254) as the validation cohort to verify the transferability and reproducibility of the prognostic role of INHBB in GC. The results suggested that patients with increased INHBB expression in the three datasets had shorter OS (Figures 3K–M).

In addition, based on the patient clinical data from GC tissue microarray, Kaplan–Meier survival analysis and log-rank statistical test were used to validate the correlation between INHBB expression levels and OS in patients with GC. Surprisingly, the same result was obtained: high INHBB expression portends a poor OS in patients with GC (Figure 5E).

### Higher inhibin subunit beta B expression is an independent prognostic factor in gastric cancer

**FIGURE 4:**
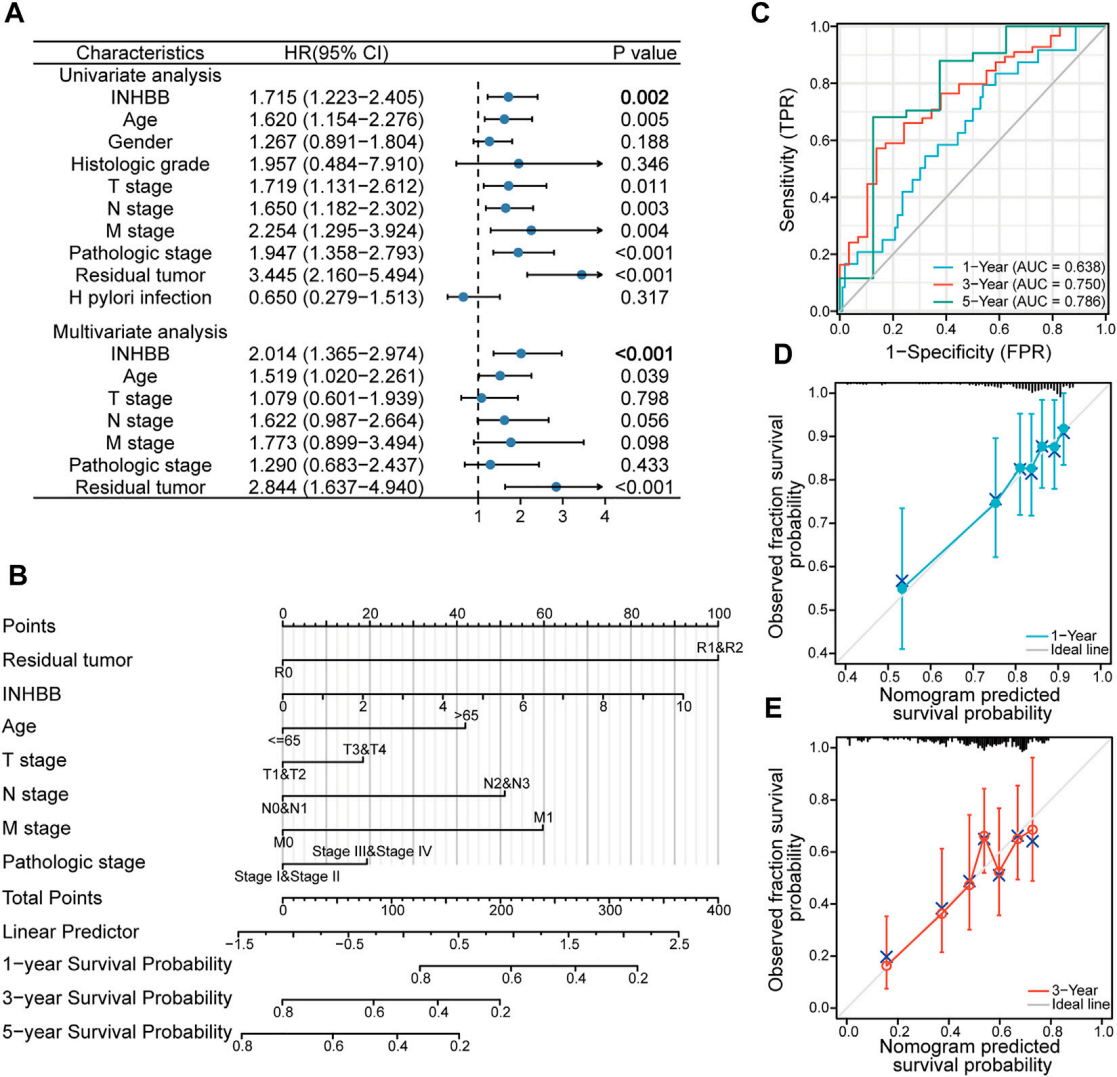
Clinical risk prediction models of INHBB in GC. **(A)** Cox regression analysis of INHBB in GC patient data from TCGA, **(B)** nomogram model based on Cox regression analysis, **(C)** Time-dependent analysis of ROC curve to evaluate nomogram performance, **(D,E)**1-year and 3-year nomogram calibration plot.

To explore the association between INHBB expression and clinical features, we conducted Cox regression analyses. The univariate Cox analysis showed that high INHBB expression is significantly related to OS (*p* = 0.002) (Figure 4A). Then, we further analyzed the data using multivariate Cox analysis and found that INHBB expression had a significant relevance with OS (HR = 2.014, 95% CI = 1.365–2.974, *p* < 0.001), as well as age (HR = 1.519, 95% CI = 1.020–2.261, *p* = 0.039) and residual tumors (HR = 2.844, 95% CI = 1.637–4.940, *p* < 0.001) (Figure 4A); these results suggested that these three indicators may be independent risk factors in GC. Moreover, we constructed a nomogram prediction model based on the results of Cox analyses, indicators included in the model include residual tumor, INHBB, age, TNM stage, and pathologic stage. The C-index of the nomogram model was 0.663 indicating a good prediction ability, and the figure showed that the prognostic prediction value of INHBB was better than that of the age and TNM stage, both of which are classical traditional clinical indicators (Figure 4B). Subsequently, we evaluated the prediction ability and agreement of this prediction model using time-dependent receiver operating characteristic (ROC) and calibration curves. The 1-, 3-, and 5-year area under the curve (AUC) values of the nomogram model were 0.638, 0.75, and 0.786 (Figure 4C). Moreover, the calibration plots of the nomogram showed an excellent agreement in the 1- and 3-year OS rates compared with that of the ideal model (Figures 4D, E). These data indicated that INHBB plays a key role in the prognosis of GC.

### Validation of inhibin subunit beta B expression and investigation of its prognostic role using gastric cancer tissue microarray

**FIGURE 5:**
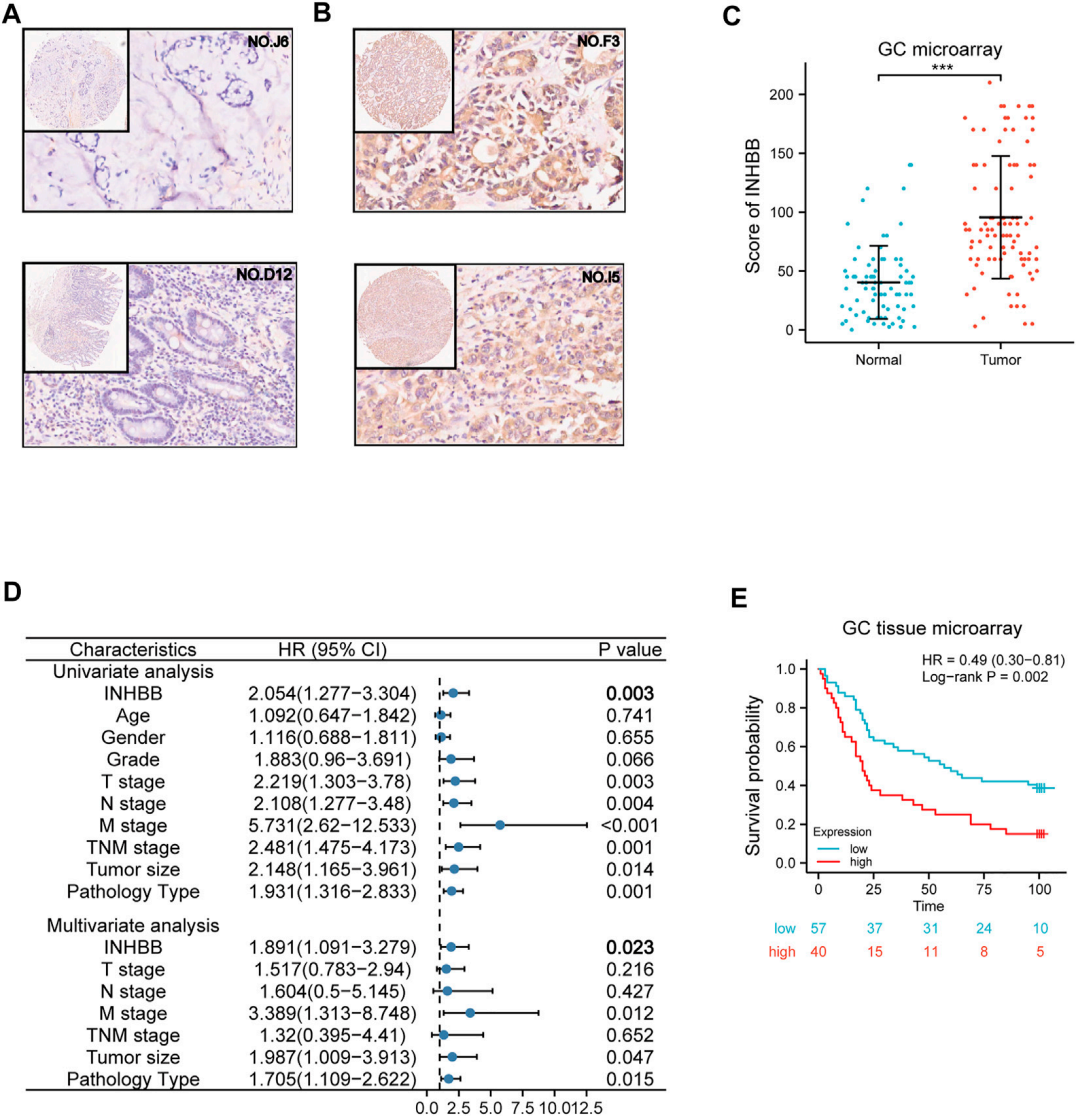
Validation of the role of INHBB in GC. **(A)** IHC of normal tissues (4X and 20X scopes), **(B)** IHC of GC tissues (4X and 20X scopes), **(C)** differential expression of INHBB in GC tissues and normal tissues, **(D)** Cox regression analysis of INHBB and clinical features in GC, **(E)** Kaplan-Meier survival analyses of INHBB in GC patient data form GC tissue microarray.

To further elucidate the role of INHBB in GC, we performed the expression and prognosis analysis of INHBB using a GC tissue microarray. First, we explored the protein expression level of INHBB in GC and normal tissues by IHC. The results indicated that the protein expression level of INHBB in GC tissues was higher than that in paracarcinoma tissues (*p* < 0.001) (Figures 5A–C). In addition, we performed univariate and multivariate Cox analyses using clinical microarray data and validated the results that high INHBB expression could be an independent risk factor in GC (Figure 5D). Kaplan–Meier survival analysis showed that patients with GC with high expression of INHBB have a worse prognosis, which is consistent with the results mentioned above (Figure 5E). These results showed that upregulated INHBB expression is associated with an adverse prognosis in GC.

### Functional and pathway enrichment analysis

**FIGURE 6:**
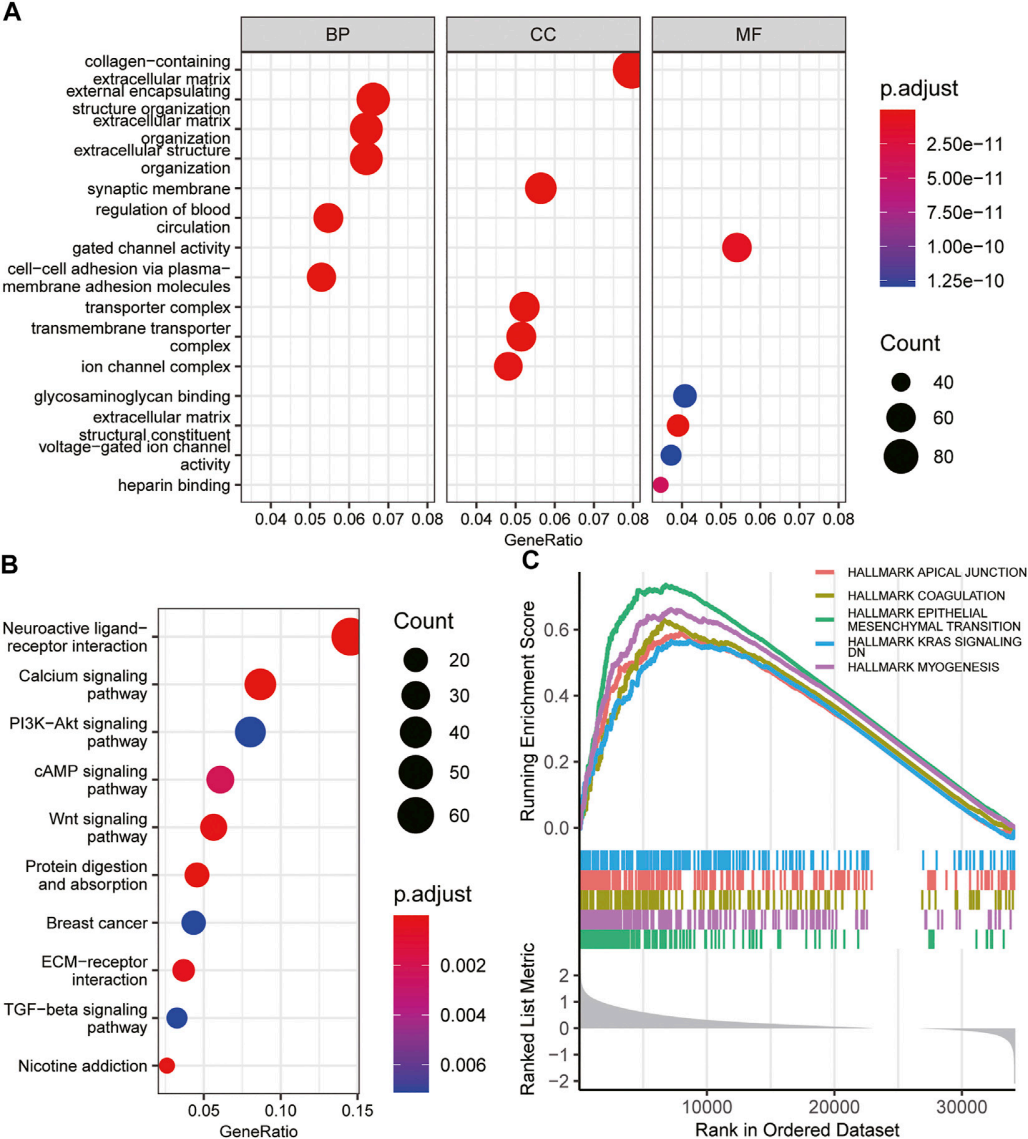
Functional enrichment of INHBB in GC. **(A)**GO enrichment analysis of INHBB in GC, **(B)** KEGG pathway analysis of INHBB in GC, and **(C)** GSEA enrichment analysis of INHBB in GC.

To elucidate the potential biological functions of INHBB in GC, GO and KEGG enrichment analysis was performed using the R software. The results indicated that the most significant terms for GO enrichment were collagen−containing extracellular matrix, external encapsulating structure organization, extracellular matrix organization, synaptic membrane, and gated channel activity (Figure 6A). The results of KEGG enrichment analysis indicated that INHBB-associated genes were mostly enriched in the following pathways: calcium signaling pathway, PI3K-Akt signaling pathway, cAMP Signaling pathway, and Wnt signaling pathway (Figure 6B).

In addition, GSEA was performed on HALLMARK gene set terms and five pathways, including hallmark epithelial-mesenchymal transition, myogenesis, coagulation, KRAS signaling, and apical junction were identified as significantly enriched (Figure 6C). These results indicated that INHBB plays a key role in cancer promotion through multiple mechanisms.

### Correlation of inhibin subunit beta B expression and immune infiltration

**FIGURE 7:**
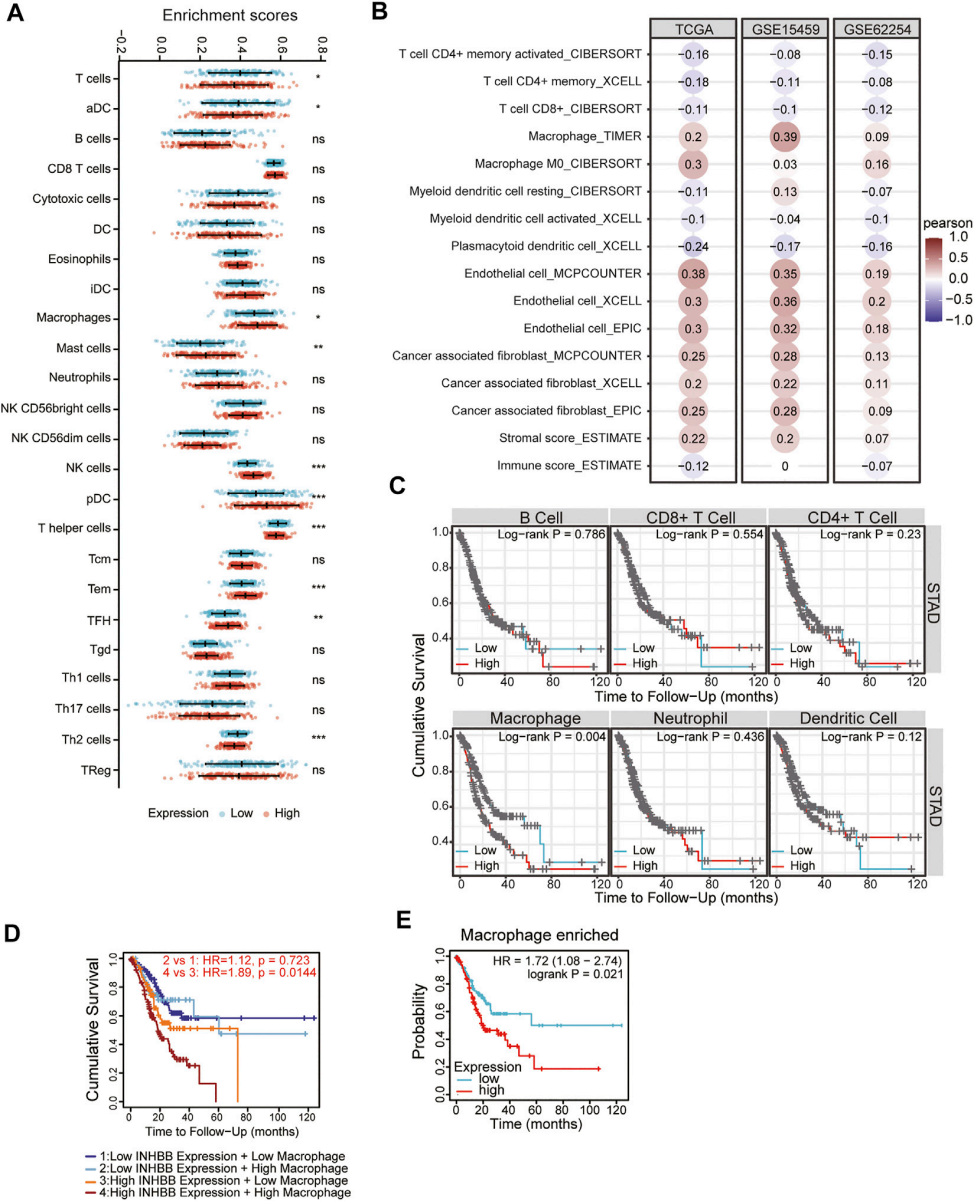
Correlation between immune infiltration and INHBB expression in GC. **(A)** INHBB expression and infiltration of 24 immune cell subtypes, **(B)** INHBB expression and immune infiltration data from TCGA and GEO, **(C)** Survival analysis for immune infiltrates and INHBB expression, **(D)** Survival analysis for differential expression of INHBB and macrophage infiltration, **(E)** Survival analysis for INHBB expression and macrophage enriched infiltration.

In the tumor microenvironment, different immune infiltration levels were significantly correlated with OS in patients with tumors. The findings mentioned above indicated that INHBB has a significant impact on the prognosis of patients with GC. Therefore, estimating the association between the expression of INHBB and immune infiltration level is reasonable. We first investigated the correlation between INHBB expression and infiltration level of 24 immune cell subtypes by single-sample GSEA (ssGSEA) (Figure 7A) and observed a significant correlation between INHBB expression and infiltration of immune cells such as Th2 cells (*p* < 0.001), NK cells (*p* < 0.001) and Tem (*p* < 0.001). In addition, we evaluated the association between the immune infiltration level and INHBB expression by various methods. The results suggested that INHBB expression was significantly correlated with macrophages (R = 0.2, p = 2e-05), endothelial cell (R = 0.38, *p* < 2.2e-16) and cancer-associated fibroblast (R = 0.25, *p* < 9.3e-08) in GC data from TCGA (Figure 7B, Supplementary Figure S6), these results were also validated using GSE15459 and GSE62254 datasets (Figure 7B). Furthermore, we further performed Kaplan-Meier analysis for immune infiltrates to visualize the survival difference in GC. As the figure shows, macrophage infiltration significantly correlated with GC prognosis (*p* = 0.004, Figure 7C). Thus, patients with GC have a worse prognosis with high INHBB expression and high macrophage infiltration compared with those with low macrophage infiltration (HR = 1.89, *p* = 0.0144, Figure 7D). Using the Kaplan–Meier plotter, we validated that high INHBB expression and macrophage-enriched infiltrates affect the prognosis of patients with GC (Figure 7E). Particularly, these results remind us whether the polarization of macrophages will affect the prognosis of patients with GC, and further analysis indicated that high macrophage M2 infiltration in patients with GC with a high or low expression of INHBB may lead to a worse survival, but the results were statistically insignificant (Supplementary Figure S7). The analysis by TIMER (Table 2, Supplementary Table S1) and GEPIA (Table 3) database also showed a strong association between INHBB expression and several markers of immune cells, especially with macrophage infiltration. All these data suggested that INHBB plays a regulatory role in immune cell infiltration, especially in the macrophage infiltration of patients with GC.

**TABLE 2:** Correlation analysis between INHBB and macrophage associated markers in TIMER.

Cell type	Gene marker	None		Purity	
		Cor	p	Cor	p
Macrophage	CD68	0.013	0.79	0	0.992
	ITGAM	0.122	*	0.119	*
M1	NOS2	−0.117	*	−0.125	*
	ROS	0.138	**	0.149	**
	IRF5	0.169	***	0.168	**
	PTGS2	0.133	**	0.141	**
M2	ARG1	0.013	0.786	0.025	0.622
	MRC1	0.094	0.056	0.088	0.087
TAM	CCL2	0.234	***	0.234	***
	CCR5	0.002	0.973	−0.008	0.884
	CD80	−0.04	0.412	−0.038	0.465
	CD86	0.056	0.252	0.055	0.283
Monocyte	CD14	0.138	**	0.13	*
	CD16	0.048	0.327	0.05	0.331
	CD115	0.159	**	0.148	**

**TABLE 3:** Correlation analysis between INHBB and macrophage associated markers in GEPIA.

Cell type	Gene marker	Tumor		Normal (TCGA)		Normal (TCGA + GTEx)	
		Cor	p	Cor	p	Cor	p
M1	NOS2	−0.12	*	0.36	*	0.28	***
	ROS	0.16	**	−0.003	0.99	−0.1	0.15
	IRF5	0.16	**	−0.21	0.23	0.13	0.056
	PTGS2	0.15	**	0.63	***	0.42	***
M2	ARG1	0.082	0.097	0.16	0.34	0.27	***
	MRC1	0.074	0.13	0.55	***	0.34	***
TAM	CCL2	0.22	***	0.38	*	0.32	***
	CCR5	0.029	0.56	−0.26	0.13	−0.14	*
	CD80	−0.051	0.31	−0.25	0.14	0.12	0.082
	CD86	0.032	0.51	−0.13	0.46	0.17	*
Monocyte	CD14	0.12	*	0.23	0.18	0.32	***
	CD16	0.033	0.51	0.091	0.6	0.38	***
	CD115	0.13	**	0.17	0.32	0.025	0.72

**FIGURE 8:**
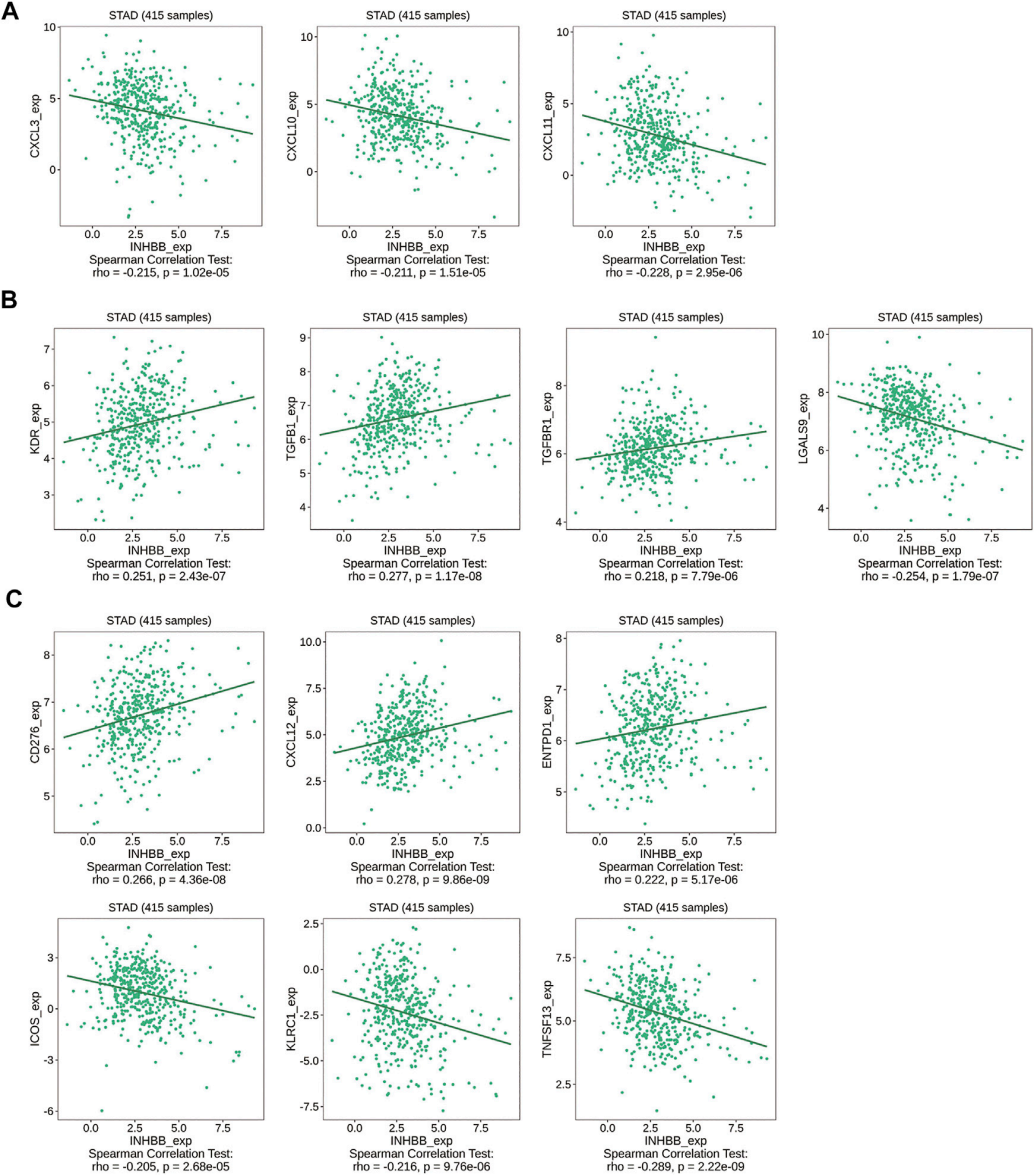
Correlation of INHBB expression and **(A)** chemokines/chemokine receptors, **(B)** immunoinhibitors, **(C)** immunostimulators.

Immune cell trafficking into the tumor microenvironment is mediated by chemokine/chemokine receptors (Nagarsheth et al., 2017). Therefore, we explored the association between INHBB expression and chemokines/chemokine receptors using the TISIDB database. The results of the heatmap demonstrated a significant correlation between several chemokines/chemokine receptors and INHBB expression in pan-cancers (Supplementary Figures S8A, B). In order to identify the association between INHBB expression and immune cell migration in GC, we further explored the association between INHBB expression and chemokines/chemokine receptors. The results suggested that INHBB expression was negatively correlated with CXCL3 (*r* = −0.215, *p* = 1.02e-05), CXCL10 (*r* = −0.211, *p* = 1.51e-05), and CXCL11 (*r* = −0.228, *p* = 2.95e-06) (Figure 8A). These results suggested a negative correlation between the expression of INHBB and chemokines/chemokine receptors.

Immunotherapy for cancer is currently thriving and immune-checkpoint blockade is a new treatment option for cancers. Therefore, we explored the association between the expression of INHBB and immunoinhibitors/immunostimulators in several cancer types (Supplementary Figures S8C, D). The results indicated that INHBB expression were positively correlated with immunoinhibitors such as KDR (*r* = 0.251, *p* = 2.43e-07),TGFB1(*r* = 0.277, *p* = 1.17e-08), and TGFBR1(*r* = 0.218, *p* = 7.79e-06), whereas it was negatively correlated with LGALS9 (*r* = −0.254, *p* = 1.79e-07) (Figure 8B); as for immunostimulators, INHBB expression were positively associated with CD276 (*r* = 0.266, *p* = 4.36e-08), CXCL12 (*r* = 0.278, *p* = 9.86e-09),ENTPD1 (*r* = 0.222, *p* = 5.17e-06) and negatively correlated with that of ICOS(*r* = −0.205, *p* = 2.68e-05), KLRC1 (*r* = −0.216, *p* = 9.76e-06), and TNFSF13(*r* = −0.289, *p* = 2.22e-09) (Figure 8C). These results suggested that INHBB plays a regulatory role in tumor immunity.

## Discussion

GC remains on the top list of cancers accounting for the highest number of deaths in the world (Bray et al., 2018). More than 30% of patient recurrence occurs within 5 years after treatment with chemotherapy, radiotherapy, and surgical resection, indicating that GC is a heterogeneous disease (Sohn et al., 2017). Protein glycosylation is a common modification occurring post-translationally in all animals with more than 90% of cell-surface proteins and lipid glycosylation helps to generate post-genomic diversity (Sweeney et al., 2018). Glycosylation alteration has been reported in several cancer types. As glycoproteins may be secreted or shed into the circulation, they can be regarded as potential biomarkers (Silsirivanit, 2019). These aberrantly expressed glycoproteins, including MUC1, MUC4, and MUC13 play important roles in tumor progression and treatment and are commonly referred to as tumor-related glycoproteins (Dhanisha et al., 2018). INHBB is a glycoprotein belonging to the TGF-β family. Recently, it has been reported that INHBB affects the development and prognosis in different tumors; however, there are few reports on the role of INHBB in GC. In the present study, we first explored the expression of INHBB using TCGA and GEO databases and discovered that INHBB was more highly expressed in GC tissues than in normal tissues, and the upregulated INHBB expression correlated with worse prognosis in patients with GC. In addition, upregulated INHBB expression was found to be associated with poor clinicopathological features. Surprisingly, all these results were validated by our clinical patient data, suggesting that INHBB could be a novel prognostic biomarker for patients with GC.

Genetics and epigenetics play a crucial role in regulating cancer progression and tumor cell evasion (Chakravarthi et al., 2016). Historically, genetic alterations such as copy number alterations and somatic mutations have been used to assess tumor evolution; however, with the advancement of research in genetic alterations, several studies showed that cell lines with a high degree of genetic homogeneity are accompanied by an increased rate of cell-to-cell variability due to epigenetic alterations (Mazor et al., 2016). Analysis of genetics and epigenetics alterations is, therefore, helpful to understand the role of gene expression in cancer progression. Subsequently, we conducted the analysis of INHBB expression by cBioPortal and found that INHBB somatic mutations and amplifications were found to be more frequent than deletions in GC. Methylation is the best studied epigenetic modification, and in general, methylation of CpG sites in the promoter is thought to silence the expression of the binding transcription factors. As expected, we found that the expression of INHBB was negatively associated with the methylation level in GC. However, it is worth noting that we only analyzed the correlation between INHBB expression and methylation sites, and whether the change in INHBB expression in GC is related to its own methylation modification still needs to be verified by subsequent experiments. Taken together, these results suggested that genetics or epigenetics alterations may affect the expression of INHBB and thus promote the progression of GC.

The tumor microenvironment is crucial for tumor progression; it contains extracellular matrix (ECM), fibroblasts, vasculature, and immune cells (Ahmed et al., 2016). ECM surrounds the tumor cells and supports their growth, survival, and eventually invasive capacity (Gordon-Alonso et al., 2017). The GO analysis showed that the structural component of ECM is the main biological function of INHBB-associated genes in GC. In addition, ECM-receptor interaction was the major enriched pathway according to the results of KEGG analysis, suggesting INHBB expression may have a specific correlation with the tumor microenvironment in GC. The other pathways observed in the KEGG analysis were involved in tumor development and progression. Ma et al. (2020) found that an imprinted gene regulates the function of cellular autophagy and epithelial–mesenchymal transition (EMT) via the PI3K/Akt pathway, thereby accelerating the deterioration progress of colorectal cancer. Calcium and cAMP are essential secondary messengers in signaling pathways in cells and both of them are central to tumorigenesis. EPAC1, one of the major downstream effectors of cAMP, regulates the metastatic properties such as proliferation and migration in triple-negative BRCA cells (Kumar et al., 2018). Similarly, the calcium signaling pathway has been found to mediate apoptosis and proliferation in non-small cell lung cancer cells (Wang et al., 2020). Moreover, pathway crosstalk of Wnt/calcium has been involved in the regulation of cell migration in cancer progression (Chuderland and Seger, 2008). Subsequently, the results of GSEA based on Hallmark gene set terms validated the oncogenetic property of INHBB in GC. There is a consensus on the key role of EMT and KRAS in cancers. Interestingly, the activation of coagulation correlated with tumor progression and invasion has been reported (Palumbo, 2008; Kvolik et al., 2010), and the potential mechanism may be related to the leaky tumor vasculature-enabling clotting factors in the blood entering the stroma (McEachron et al., 2010). Our data showed that INHBB is an oncogene and accelerates the development of GC in multiple ways.

As mentioned above, the tumor microenvironment is crucial for cancer progression and immune cells are key players in it. Previous studies have reported that immune cell infiltrates within a tumor are associated with disease prognosis and response to immunotherapy (Buisseret et al., 2018). Therefore, it is necessary to clarify the relationship between INHBB expression and immune infiltration in GC. In this pilot study, we identified that INHBB expression is positively correlated with the infiltration of macrophages, endothelial cells, and cancer-associated fibroblasts and is negatively associated with the infiltration of CD4+/CD8+ T cells and plasmacytoid dendritic cells. Further analyses showed that macrophage infiltration has an important impact on the prognosis of patients with GC. In tumors, macrophages are differentiated from monocytes and usually divided into two subtypes: M1, antitumor macrophages, and M2, tumor-promoting macrophages. Particularly, macrophage infiltration in tumors is heightened in the M2 subunit and not M1 (Mantovani et al., 2002). In turn, these macrophages foster a suitable microenvironment that supports tumor cell survival. An increased level of macrophage infiltration in tumors portends a worse prognosis (Qian and Pollard, 2010). Not surprisingly, our results showed that both the increased macrophage infiltration with upregulated INHBB expression correlated with poor prognosis in GC. Moreover, we found that high infiltration of M2 macrophages predicts worse survival, although the findings were not statistically significant. These findings suggested that INHBB may regulate the microenvironment of immune cell infiltration to promote GC progression.

Since chemokines secreted by tumor cells recruit immune cells to the tumor sites, we explored the association between INHBB expression and expression of chemokines/chemokine receptors in GC. The results suggested that the expression of CXCL3, CXCL10, and CXC11 were negatively correlated with INHBB expression in GC. CXCL3 is a known angiogenic chemokine and participates in the chemotaxis of neutrophils (Rainard et al., 2008), in addition, CXCL3 inhibits the growth of esophageal squamous cell carcinoma by attracting neutrophils (Chen et al., 2017). CXCL10 is induced by IFNγ and is responsible for the recruitment of T cells into tumors. Jiang, Zheng et al. found that the down-regulated expression of CXCL10 was a worse prognostic indicator in colorectal cancer (Jiang et al., 2010). Similar to CXCL10, CXC11 is also a downstream target of IFNγ. In T lymphocytes, it mediates antitumor immune responses (Hensbergen et al., 2005), which supports the result of this study that a low level of CXC11 is related to poor prognosis in GC. The immune system is complicated, and immunotherapy has changed the standard of care for multiple malignant tumors; among them, blocking inhibitory immune checkpoints has become an attractive antitumor strategy. Immunostimulators play an important role in immune activation, and the overexpression of immunoinhibitors is a clinical anti-checkpoint or combination treatment strategy. In this study, we explored the association between INHBB expression and expression of immunoinhibitors/immunostimulators and found several potential INHBB-associated targets of immunotherapy for GC. These results suggest that INHBB plays an essential role in immune infiltration in GC.

This study also has some limitations. First, the data of expression and prognosis were obtained from multiple online databases, making it difficult to ensure authenticity and integrity; however, our microarray data included complete clinical information, and the expression of INHBB was validated by experiments. Second, we found that INHBB expression is associated with immune infiltration, although the data were mostly derived from the TCGA database. We used TCGA because it is one of the most commonly used oncology research-related portals with a complete set of data; in addition, we corroborated the results with those from the GEO database.

## Conclusion

In summary, we found that INHBB was upregulated in GC tissues; high INHBB expression is an independent risk factor and is associated with poor prognosis in GC. Moreover, INHBB expression is significantly correlated with immune cell infiltration, especially macrophage infiltration. The alteration of INHBB-related immune cell infiltration may affect the prognostic outcome of GC patients, its clinical value deserves further exploration in the future.

## Data Availability

The original contributions presented in the study are included in the article/Supplementary Material, further inquiries can be directed to the corresponding authors.
