# Supplementary figures and images for: INHBB is a novel prognostic biomarker and correlated with immune infiltrates in gastric cancer
Source: Front Genet. 2022 Sep 2;13:933862. doi: 10.3389/fgene.2022.933862 (PMC9478859; doi:10.3389/fgene.2022.933862)

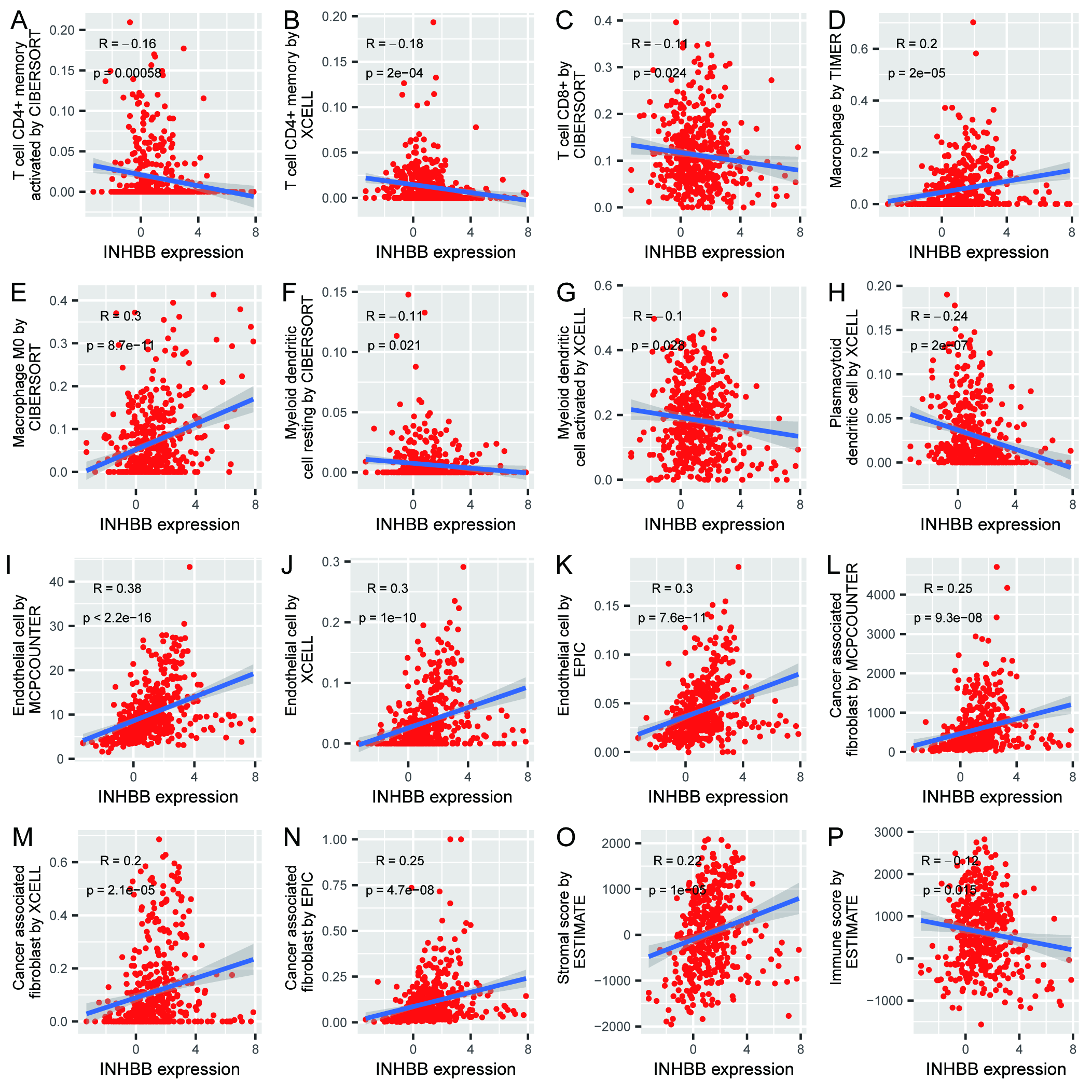

Supplement: Supplementary file 2 [file Image6.TIF]

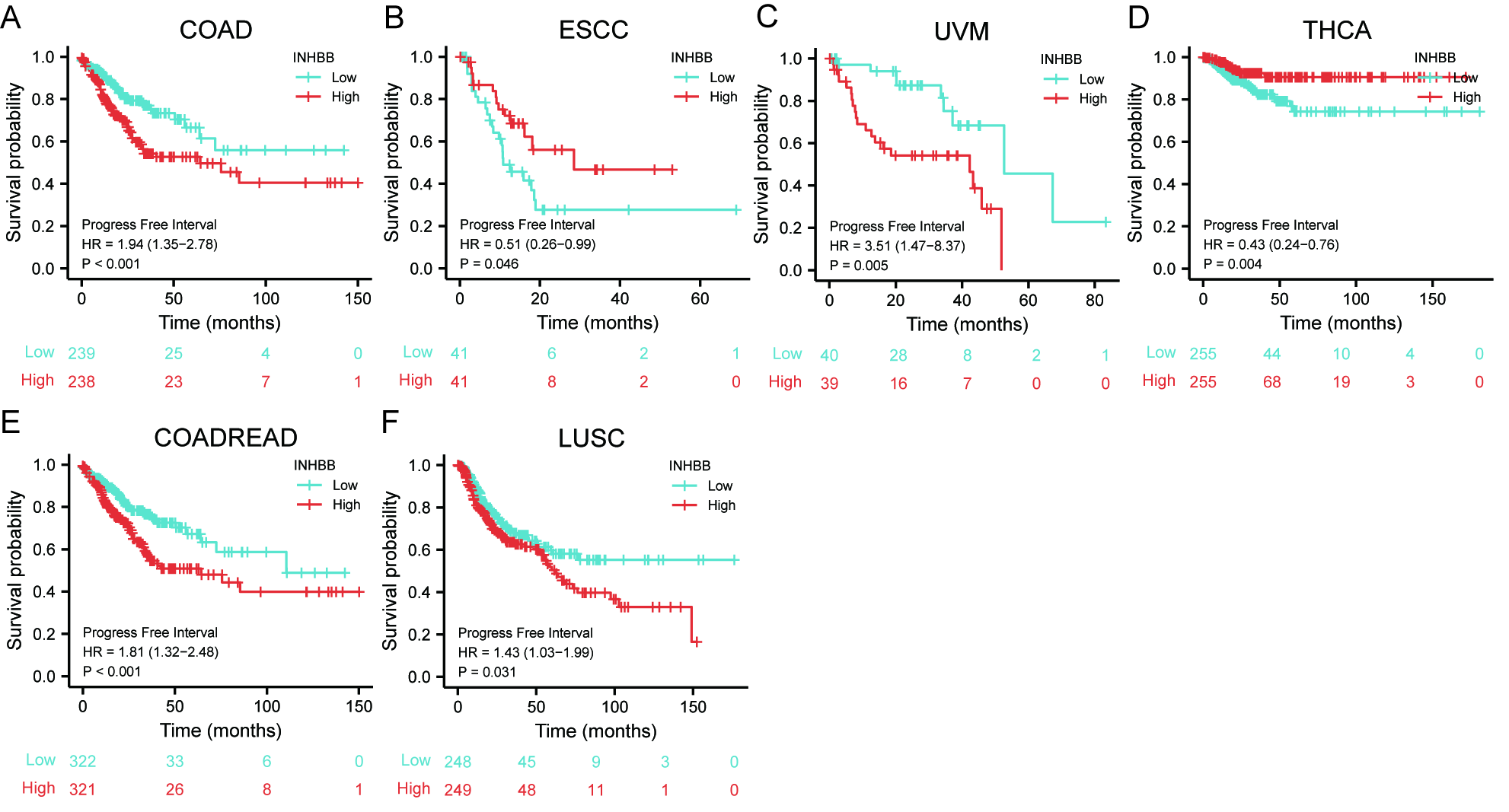

Supplement: Supplementary file 4 [file Image3.TIF]

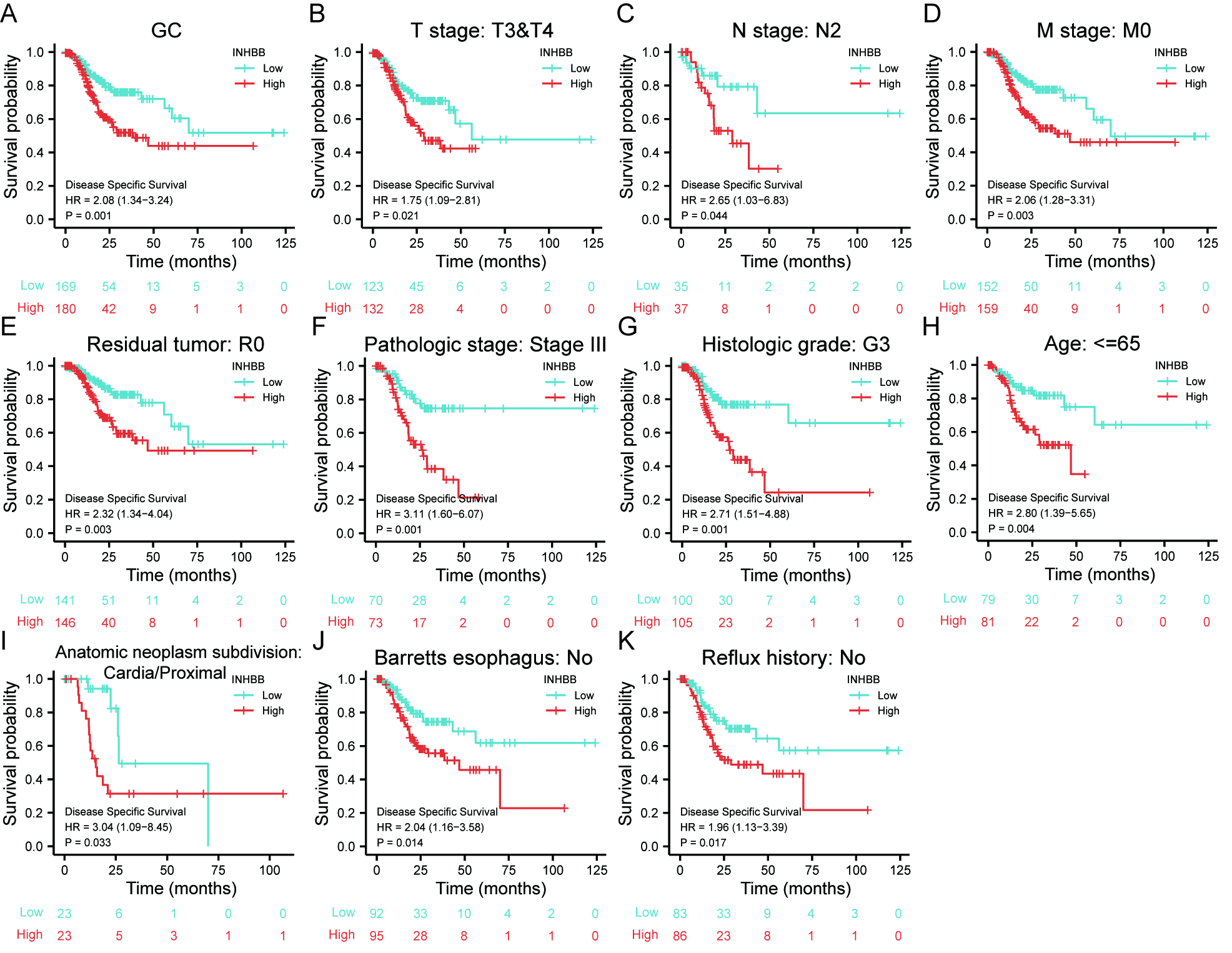

Supplement: Supplementary file 5 [file Image4.TIF]

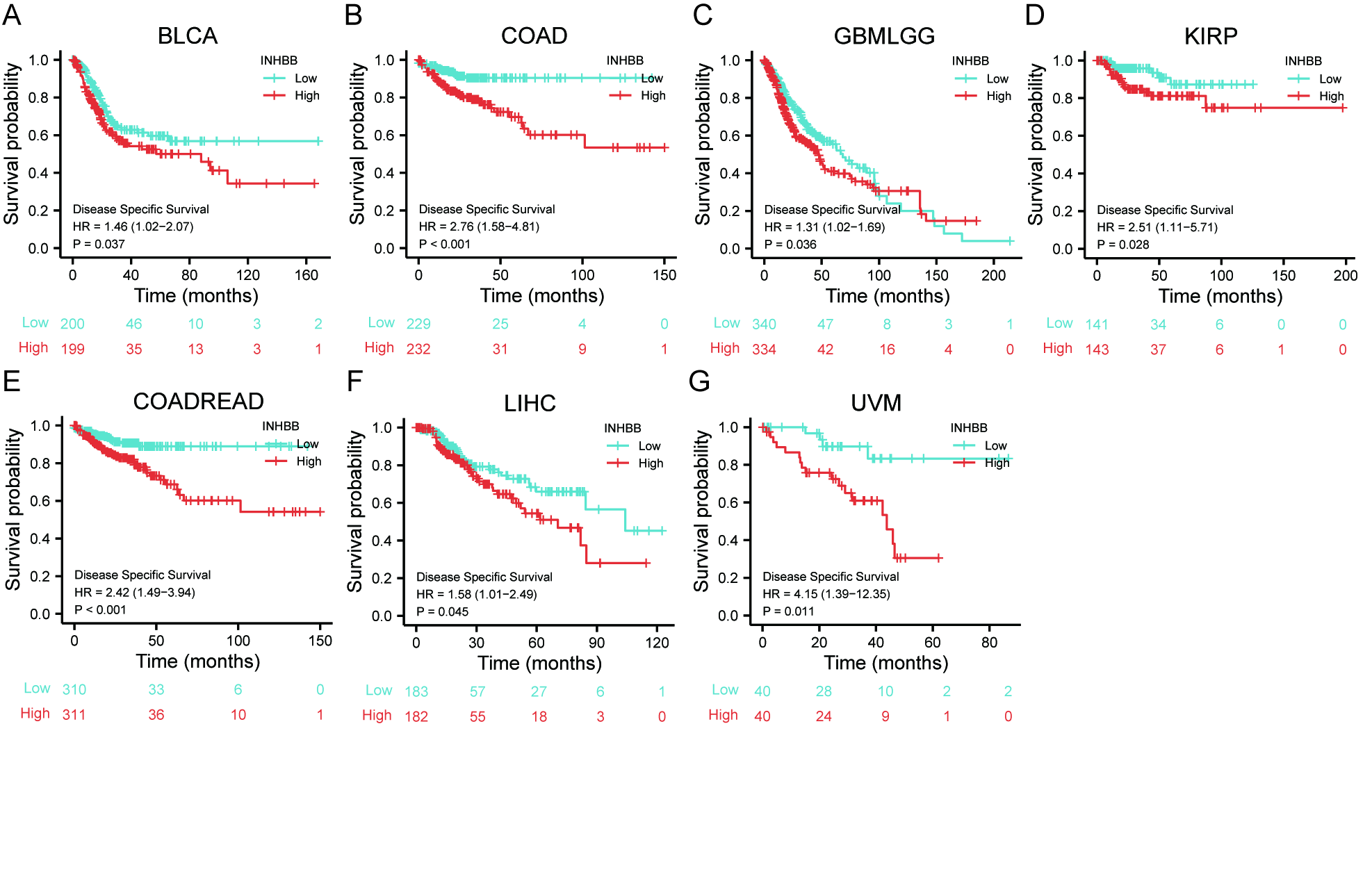

Supplement: Supplementary file 6 [file Image2.TIF]

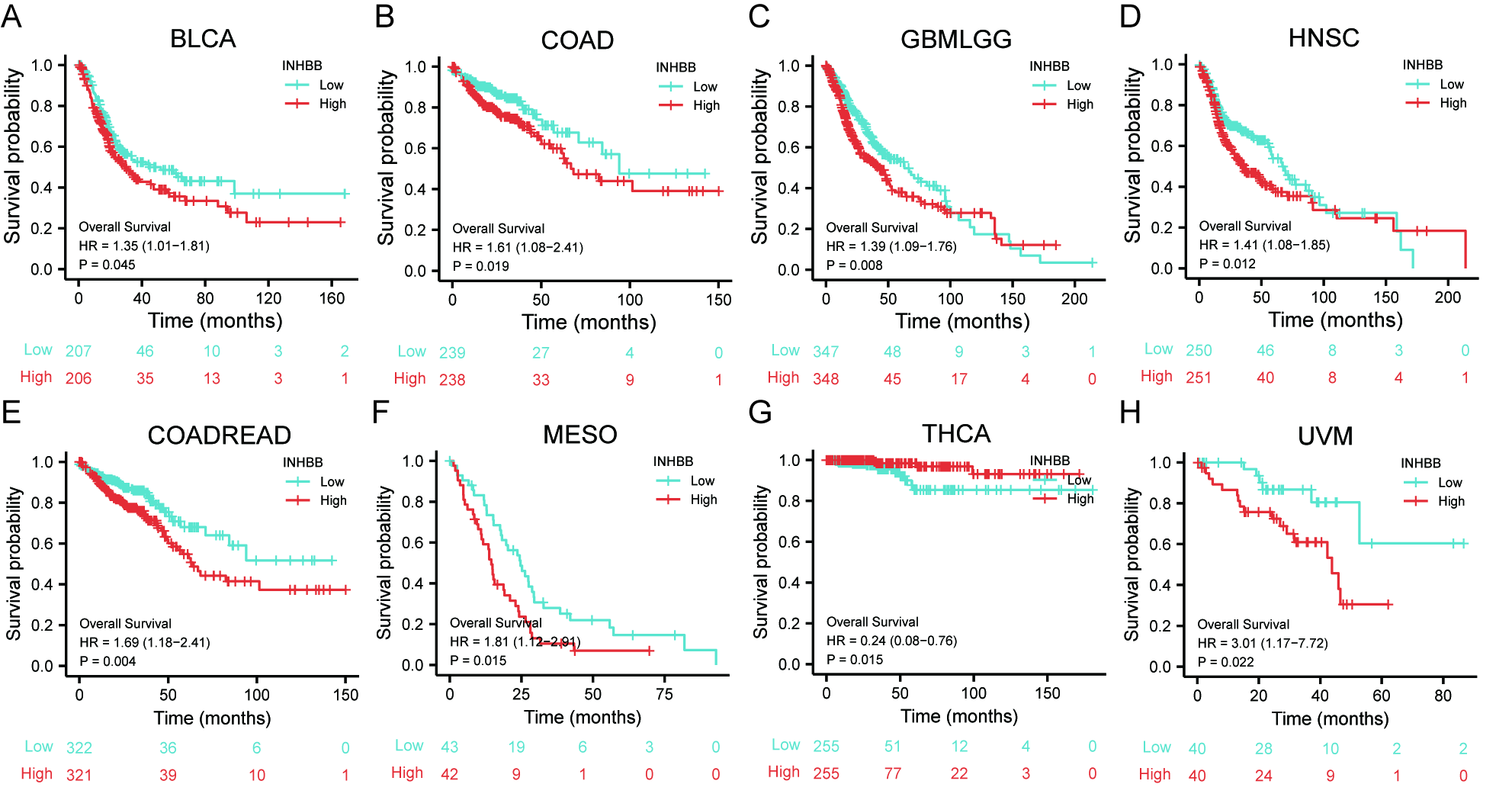

Supplement: Supplementary file 7 [file Image1.TIF]

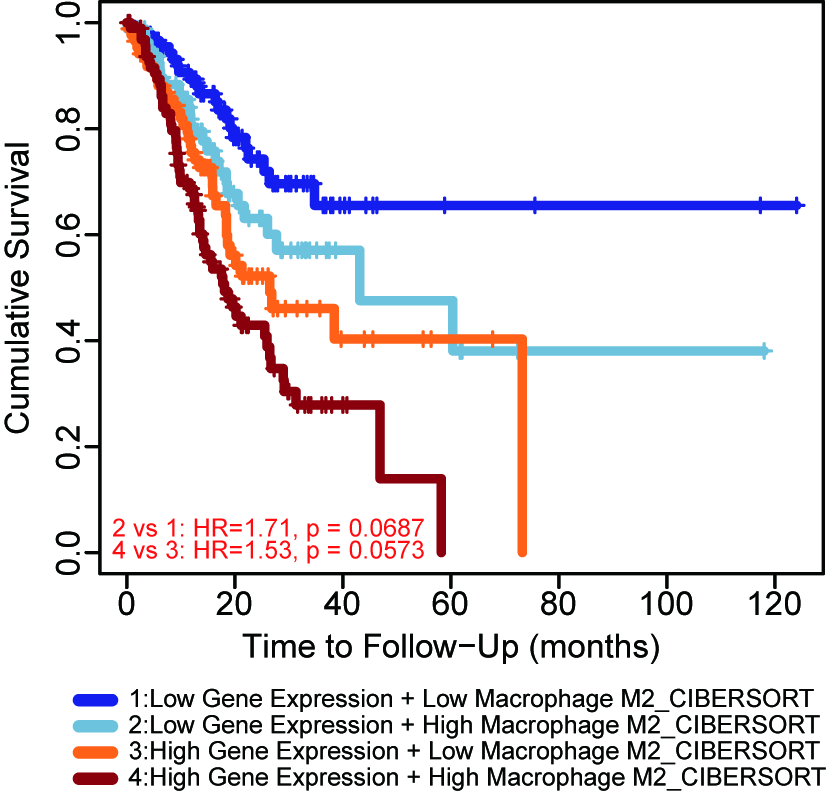

Supplement: Supplementary file 8 [file Image7.TIF]

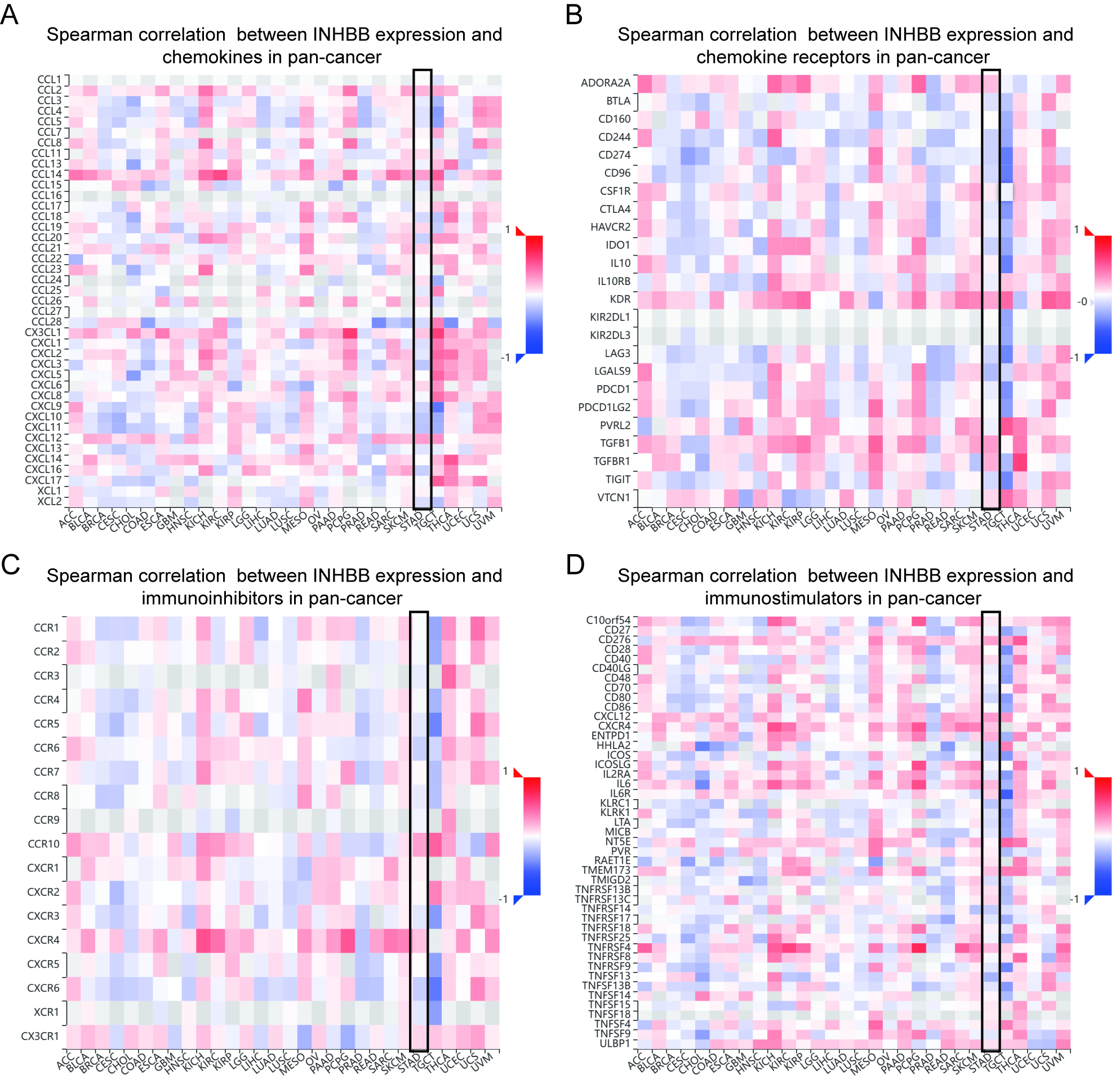

Supplement: Supplementary file 10 [file Image8.TIF]

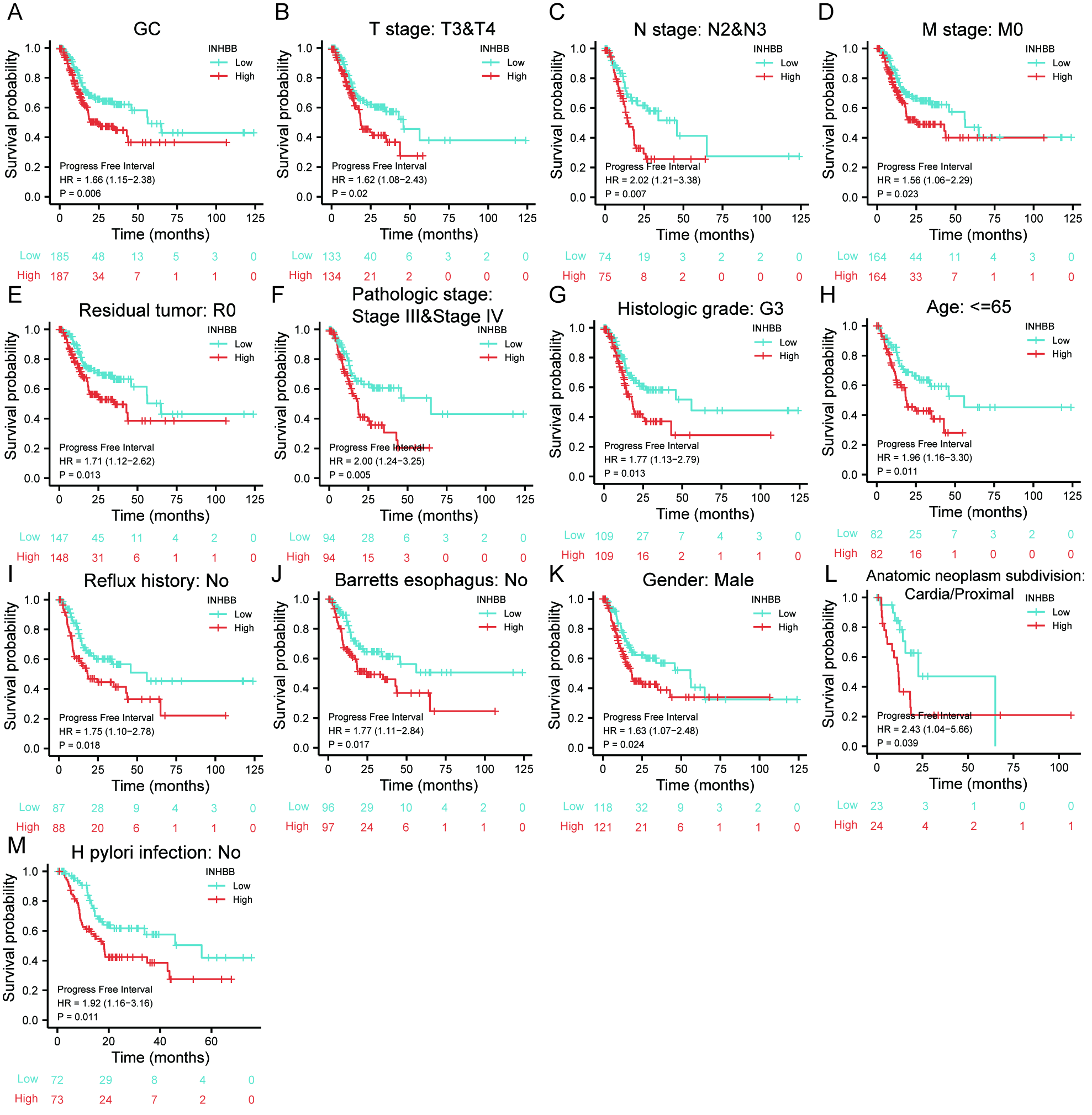

Supplement: Supplementary file 11 [file Image5.TIF]
