# Supplementary material for: INHBB is a novel prognostic biomarker and correlated with immune infiltrates in gastric cancer
Source: Front Genet. 2022 Sep 2;13:933862. doi: 10.3389/fgene.2022.933862 (PMC9478859; doi:10.3389/fgene.2022.933862)
Supplement: Supplementary file 3 [file Table1.DOCX]

**Table S1. Correlation analysis between INHBB and markers of immune cells in TIMER**

| **Cell type** | **Gene marker** | **None** |  |  | **Purity** |  |
| --- | --- | --- | --- | --- | --- | --- |
|  |  | **Cor** | **p** |  | **Cor** | **p** |
| B cell | CD19 | 0.067 | 0.171 |  | 0.067 | 0.191 |
|  | KRT20 | -0.102 | * |  | -0.125 | * |
|  | CD38 | 0.03 | 0.54 |  | 0.017 | 0.742 |
| CD8+T cell | CD8A | 0.004 | 0.942 |  | -0.01 | 0.84 |
|  | CD8B | 0.048 | 0.33 |  | 0.046 | 0.374 |
| Tfh | CXCR5 | 0.07 | 0.154 |  | 0.061 | 0.239 |
|  | ICOS | 0.086 | 0.08 |  | -0.096 | 0.061 |
|  | BCL-6 | 0.383 | *** |  | 0.386 | *** |
| Th1 | IL12RB2 | -0.019 | 0.703 |  | -0.011 | 0.83 |
|  | WSX-1 | 0.061 | 0.218 |  | 0.056 | 0.275 |
|  | STAT4 | 0.012 | 0.815 |  | 0.002 | 0.975 |
|  | IFNG | -0.199 | *** |  | -0.186 | *** |
|  | TBX21 | -0.029 | 0.56 |  | -0.028 | 0.591 |
|  | STAT1 | -0.054 | 0.268 |  | -0.043 | 0.405 |
|  | TNF-α | 0.003 | 0.956 |  | 0.008 | 0.874 |
| Th2 | CCR3 | -0.008 | 0.869 |  | -0.016 | 0.753 |
|  | STAT6 | 0.006 | 0.9 |  | -0.01 | 0.848 |
|  | GATA3 | 0.108 | * |  | 0.102 | * |
|  | STAT5A | 0.106 | * |  | 0.095 | 0.066 |
| Th9 | TGFBR2 | 0.297 | *** |  | 0.28 | *** |
|  | IRF4 | 0.028 | 0.57 |  | 0.018 | 0.726 |
|  | SPI1 | 0.094 | 0.055 |  | 0.096 | 0.063 |
| Th17 | IL-21R | 0.024 | 0.627 |  | 0.02 | 0.694 |
|  | IL-23R | -0.034 | 0.493 |  | -0.052 | 0.31 |
|  | STAT3 | 0.265 | *** |  | 0.255 | *** |
| Th22 | CCR10 | 0.303 | *** |  | 0.287 | *** |
|  | AHR | 0.048 | 0.326 |  | 0.028 | 0.589 |
| Treg | FOXP3 | 0.029 | 0.561 |  | 0.011 | 0.835 |
|  | CCR8 | 0.047 | 0.339 |  | 0.028 | 0.586 |
|  | IL2RA | 0.008 | 0.877 |  | -0.004 | 0.932 |
| T cell exhaustion | PDCD1 | 0.024 | 0.632 |  | 0.029 | 0.574 |
|  | CTLA4 | 0.022 | 0.657 |  | 0.028 | 0.585 |
|  | HAVCR2 | 0.044 | 0.376 |  | 0.043 | 0.402 |
| NK | XCL1 | 0.072 | 0.143 |  | 0.086 | 0.092 |
|  | KIR3DL1 | -0.119 | * |  | -0.134 | ** |
|  | CD7 | 0.113 | * |  | 0.116 | * |
| Neutrophil | FUT4 | -0.123 | * |  | -0.153 | ** |
|  | MPO | 0.277 | *** |  | 0.283 | *** |
|  | CEACAM8 | -0.042 | 0.392 |  | -0.031 | 0.542 |
|  | ITGAM | 0.122 | * |  | 0.119 | * |
| DC | BDCA1 | 0.091 | 0.065 |  | 0.089 | 0.082 |
|  | THBD | 0.415 | *** |  | 0.404 | *** |
|  | ITGAX | 0.138 | ** |  | 0.139 | ** |
